# *erm*(T)-Mediated Macrolide-Lincosamide Resistance in Streptococcus suis

**DOI:** 10.1128/spectrum.01657-21

**Published:** 2022-01-12

**Authors:** Rui Yu, Yindi Xu, Stefan Schwarz, Yanhong Shang, Xuezhen Yuan, Yue Zhang, Dexi Li, Xiang-Dang Du

**Affiliations:** a College of Veterinary Medicine, Henan Agricultural University, Zhengzhou, People’s Republic of China; b Institute of Animal Husbandry and Veterinary Research, Henan Academy of Agricultural Sciences, Zhengzhou, People’s Republic of China; c Institute of Microbiology and Epizootics, Centre for Infection Medicine, Department of Veterinary Medicine, Freie Universität Berlin, Berlin, Germany; d Henan Institute of Veterinary Drug and Feed Control, Zhengzhou, People’s Republic of China; INTHERES

**Keywords:** *Streptococcus suis*, *erm*(T), resistance, macrolide, lincosamide, fitness cost

## Abstract

To investigate the presence and location of *erm*(T) in clinical Streptococcus suis isolates and explore the transmission ability and fitness cost of *erm*(T)-carrying mobile genetic elements among S. suis isolates, MICs were determined by broth microdilution. The presence of *erm*(T) in S. suis was detected by PCR. The genetic environment of *erm*(T) in S. suis was explored by whole-genome sequencing (WGS) analysis. Intraspecies and interspecies transmission were examined by electrotransformation. The fitness cost associated with the carriage of an *erm*(T)-harboring plasmid or an integrative and conjugative element (ICE) was examined by competition experiments. Of 237 nonduplicate strains, *erm*(T) was detected in 2 S. suis strains (SC262-ST954 and SC117-ST1314), with its location on a 5,125-bp plasmid in S. suis SC262 and on a 64,013-bp ICE*Ssu*SC117 in S. suis SC117, respectively. Both the *erm*(T)-carrying plasmid pSC262 and the ICE*Ssu*SC117 were transmissible by transformation. Plasmid pSC262 can replicate and express macrolide-lincosamide resistance in heterologous hosts, including S. aureus and S. pneumoniae. Both the *erm*(T)-carrying plasmid and the ICE posed a fitness cost to the host S. suis isolate. To the best of our knowledge, this is the first report of the macrolide-lincosamide-streptogramin B resistance gene *erm*(T) in S. suis. Its location on a plasmid or an ICE will aid in its transmission. The low detection rate of *erm*(T) gene among the S. suis population might be due to the fitness cost of the *erm*(T)-carrying plasmid and ICE.

**IMPORTANCE** Macrolide and lincosamide resistance due to the presence of *erm*(T) have posed a challenge for the treatment of Gram-positive pathogens. Although the low detection rate of *erm*(T) gene among the S. suis population due to the fitness cost of the *erm*(T)-carrying plasmid and ICE, the presence of *erm*(T) in S. suis and its potential transmission to other Gram-positive pathogens will be of important significance.

## INTRODUCTION

Streptococcus suis is an important Gram-positive pathogen in the swine industry and also an emerging zoonotic pathogen in humans. Antimicrobial treatment is one of the most important measures to control the S. suis infections (1, 2). Penicillins, macrolides, lincosamides, fluoroquinolones, and tetracyclines are often used as first-line treatments. However, antimicrobial resistance has been emerging during the past years (3), which poses a great challenge not only to the swine industry but also to the public health.

Macrolide resistance is commonly mediated by *erm* genes and complemented by *mef* and *msr* genes (4, 5). The *erm*(T) gene was first identified in Lactobacillus reuteri; since then, it has been described in various Gram-positive organisms, including the genera *Lactobacillus*, *Streptococcus*, *Staphylococcus*, *Enterococcus*, and *Erysipelothrix*, and also in Gram-negative organisms, such as *Glaesserella* (*Haemophilus*) *parasuis* (6–11). In most cases, the *erm*(T) gene was located on broad host-range plasmids of variable sizes. In Staphylococcus aureus, the plasmid-borne *erm*(T) gene was flanked by two copies of IS*431* elements (12), or together with other antimicrobial resistance genes, such as *tet*(L) and/or *dfrK*, flanked by two copies of IS*Sau10* (13). In addition, the *erm*(T) gene was also identified in the chromosomal DNA of Streptococcus gallolyticus subsp. *pasteurianus*, where it was flanked by two copies of IS*1216V*-like elements (14).

To date, the information about *erm*(T) in S. suis is still limited. Therefore, this study was initiated to analyze the presence and location of *erm*(T) in clinical S. suis isolates. In addition, the transferability and fitness cost of *erm*(T) among S. suis isolates were explored.

## RESULTS AND DISCUSSION

### Plasmid- and ICE-borne *erm*(T) genes were identified in S. suis.

**FIG 1:**
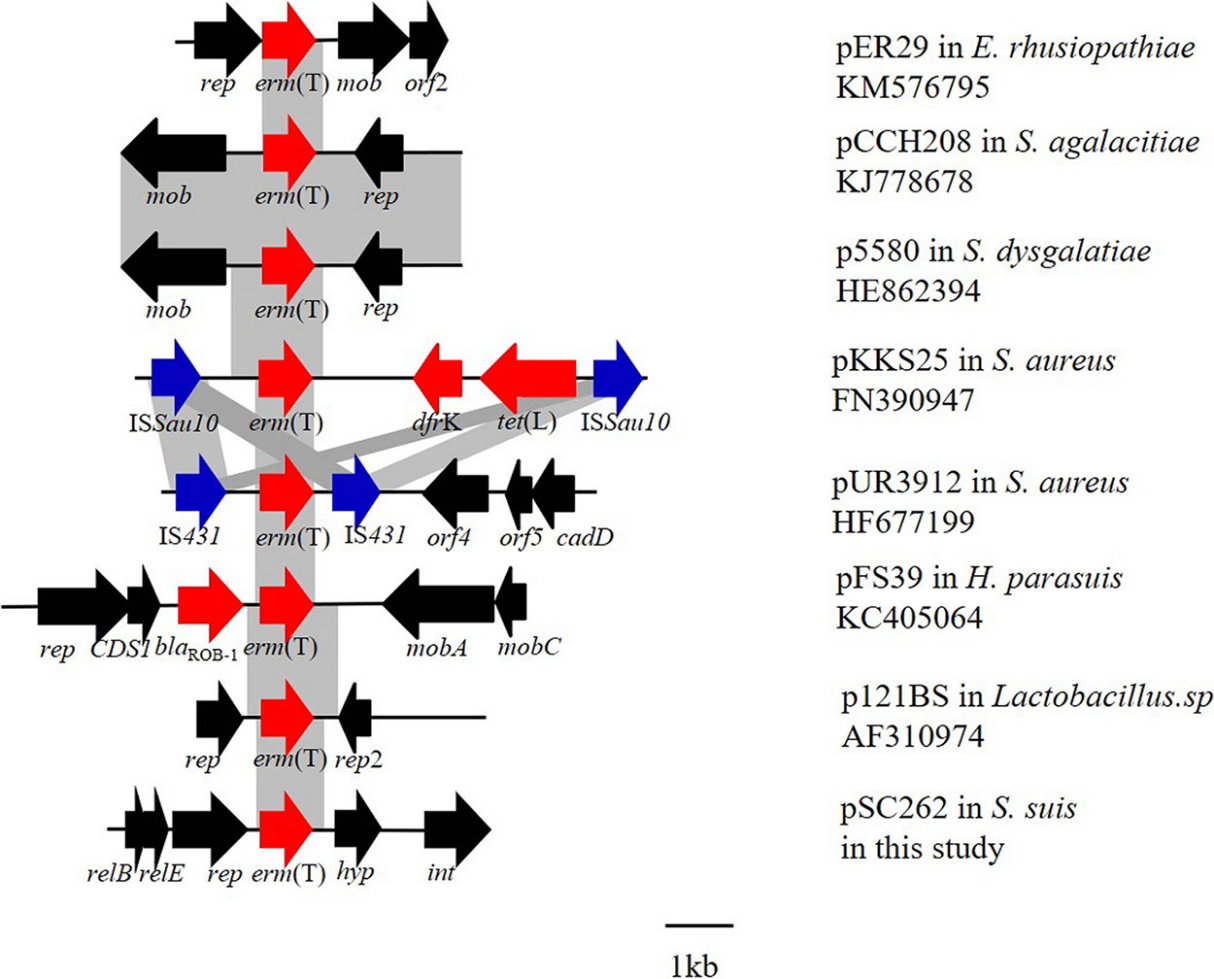
The structural comparison of the *erm*(T) gene in plasmid DNA from S. suis in this study with that from E. rhusiopathiae, S. agalactiae, S. dysgalactiae, S. aureus, G. parasuis, and *Lactobacillus* sp. Resistance genes are shown in red and other genes are shown in black.

**FIG 2:**
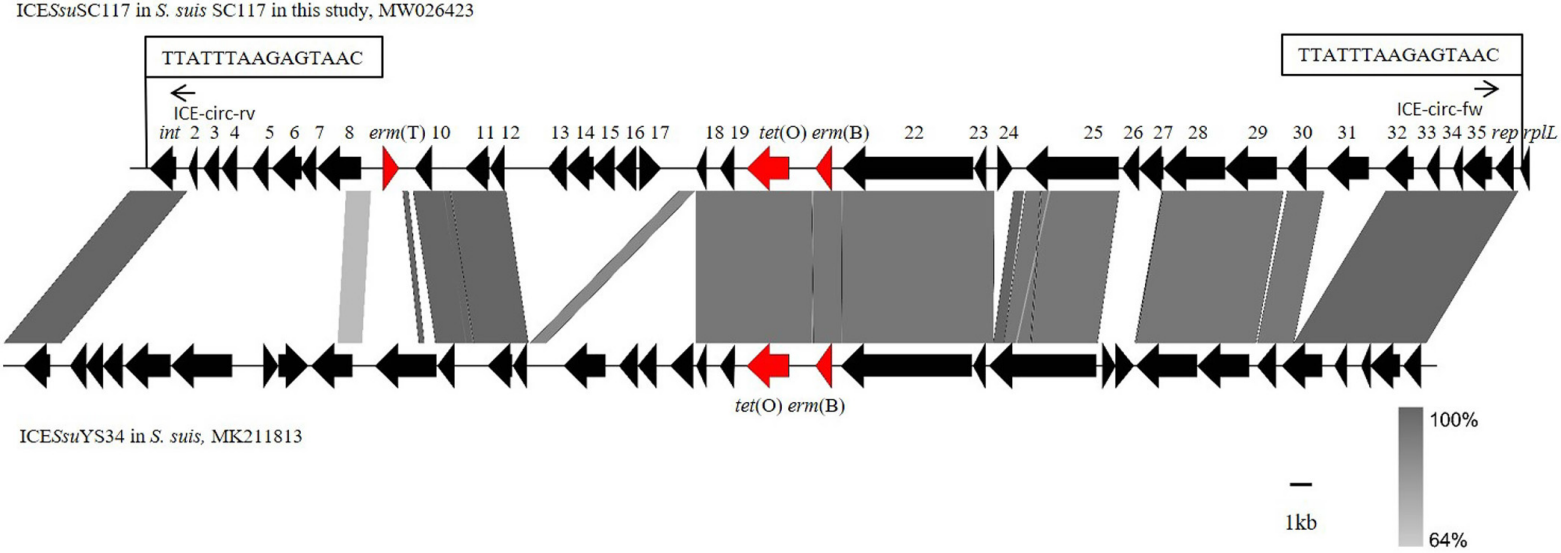
The structural comparison of *erm*(T)-carrying ICE*Ssu*SC117 in S. suis in this study with ICE*Ssu*YS34 in S. suis (MK211813) described previously. The locations of the PCR primers for the detection of circularizable forms of the ICE are indicated by arrows. The perfect 15 bp target site duplications at both termini are shown in boxes. Regions with more than 90% nucleotide sequence identity are shaded gray.

In our study, 2 *erm*(T)-positive S. suis isolates, SC262-ST954 and SC117-ST1314, out of 237 nonduplicate isolates were identified. In S. suis SC262, *erm*(T) was located on a small plasmid of 5,125 bp, which is similar to previously described *erm*(T)-carrying plasmids, such as pER29 from Erysipelothrix rhusiopathiae (KM576795), pCCH208 from S. agalactiae (KJ778678), p5580 from S. dysgalactiae (HE862394), pUR2940, pKKS25, and pUR3912 from S. aureus (HF583292, FN390947, and HF677199), pFS39 from Glaesserella parasuis (KC405064), and p121BS from *Lactobacillus* sp. (AF310974) (Fig. 1). The previous study identified a complete translational attenuator immediately upstream of the *erm*(T) gene on the plasmid pRW35 which consisted of two pairs of inverted-repeat sequences of 12 bp each and a reading frame for a regulatory peptide of 19 amino acids (aa) (15). Comparison of the *erm*(T) regulatory region of pSC262 with that of plasmid pRW35 (EU192194) revealed that the *erm*(T) regulatory region of pSC262 had many point mutations compared with that of pRW35, and only ribosomal binding sites RBS2 and IR2 could match perfectly (Fig. S1). The *rep* gene of plasmid pSC262 was compared with those deposited in NCBI GenBank, and a 58.85% identity with that of plasmid pPTDrAP from S. aureus was found, which may point toward the across-genus dissemination potential of this plasmid. As shown in Fig. 2, *erm*(T) is located on an ICE in S. suis SC117, designated ICE*Ssu*SC117, which belongs to the ICE*Sa*2603 family of ICEs and has a size of 64,013 kb. ICE*Sa*2603 is a 54-kb ICE originally found in S. agalactiae 2603V/R (16). An ICE that carries an integrase gene closely related to int_ICE_*_Sa_*_2603_, defined as having >60% gene or protein homology, and has significant sequence alignment (60% nucleic acid identity of core genes) and syntonic core structure was classified as a member of the ICE*Sa*2603 family (17). The nucleic acid homology between int_ICE_*_Ssu_*_SC117_ and int_ICE_*_Sa_*_2603_ is 94.55%. ICE*Ssu*SC117 had 95.90% identity and 55.00% coverage rate with the ICE*Sa*2603. In addition, it has a core structure similar to that of ICE*Sa*2603. Therefore, ICE*Ssu*SC117 was classified into the ICE*Sa*2603 family. The DNA sequence of ICE*Ssu*SC117 was compared with those deposited in the GenBank, and the BLASTn result indicated that it had 95.42% identity and 60.00% coverage rate with the ICE*Ssu*YS34 in S. suis (MK211813). The 65.361 kb ICE*Ssu*YS34 in the S. suis strain carried the resistance genes *erm*(B) and *tet*(O) but not *erm*(T). ICE*Ssu*SC117 was inserted at *rplL* locus, which is one of the common insertion hot spots of mobile genetic elements (MGEs) in S. suis, forming perfect 15 bp target site duplications at both termini (5′-TTATTTAAGAGTAAC-3′). The 15 bp sequence (TTATTTAAGAGTAAC) at the 3′ end of *rplL*, the insertion hot spot of ICEs, is highly conserved in streptococci (18). To verify the formation of circular ICE*Ssu*SC117 structures, specific primers (ICE-circ-fw/ICE-circ-rv) were designed, and then a 2,537 bp amplicon was detected, which confirmed the ability of ICE*Ssu*SC117 to excise from the S. suis chromosomal DNA and to form a circular intermediate. Similarly, the *erm*(T) upstream regulatory region of ICE*Ssu*SC117 was compared with that of plasmid pRW35. The results indicated that the *erm*(T) upstream regulatory region of ICE*Ssu*SC117 had 5 bp point mutations and 1 bp insertion compared to pRW35 in the regulatory peptide open reading frame (ORF). This 1 bp insertion resulted in a frameshift mutation, which extended the reading frame for the regulatory peptide from 19 aa to 28 aa (Fig. S2). The results of the test for inducible clindamycin resistance showed that S. suis strains SC262 and SC117 were resistant to both erythromycin and clindamycin, which revealed that the expression of *erm*(T) in pSC262 and ICE*Ssu*SC117 was constitutive.

### The *erm*(T) gene can be transmissible.

**FIG 3:**
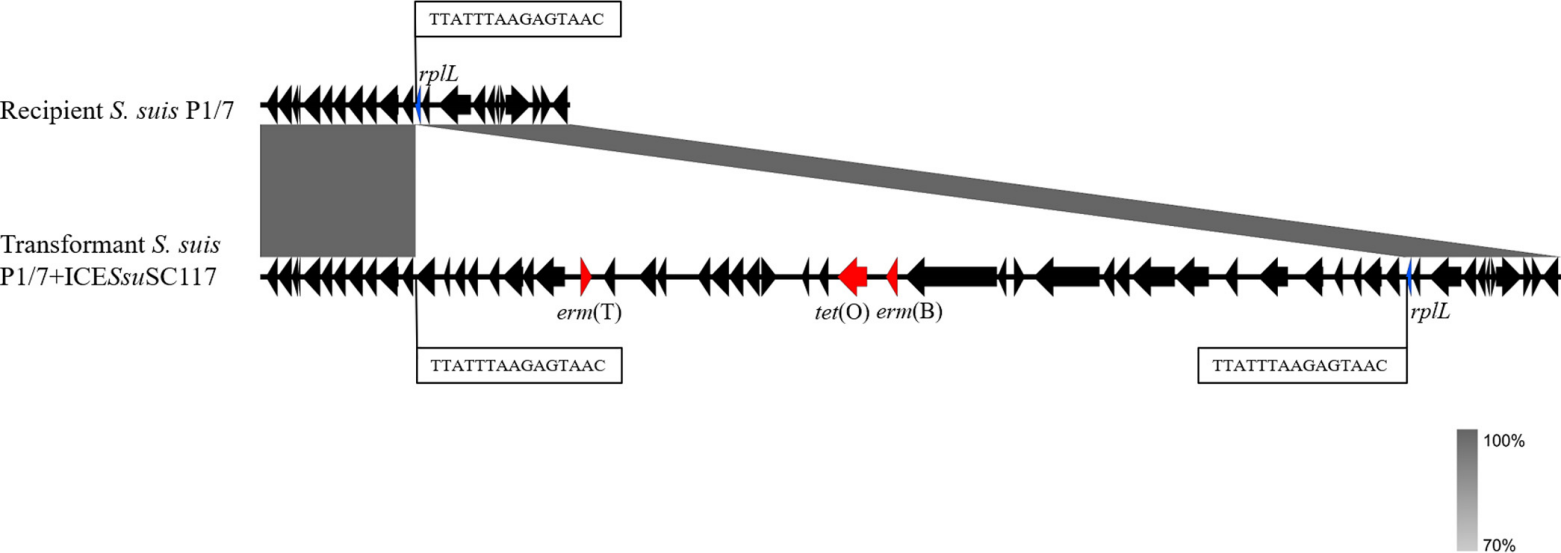
The structural comparison between the recipient S. suis P1/7 and the transformant S. suis P1/7+ICE*Ssu*SC117. The 15 bp target sites are shown in boxes. Regions with more than 70% nucleotide sequence identity are shaded gray. Resistance genes are shown in red, *rplL* genes are shown in blue, and other genes are shown in black.

Transformation experiments indicated that both the *erm*(T)-carrying plasmid pSC262 and ICE*Ssu*SC117 are transmissible. The transformants P1/7+pSC262 and P1/7+ICE*Ssu*SC117 displayed the elevated MICs to the respective antimicrobial agents compared with those of the recipient strain (Table 1). WGS analysis indicated that the *erm*(T)-carrying ICE*Ssu*SC117 was entirely integrated into the *rplL* gene in the recipient strain, with 15 bp target duplications (5′-TTATTTAAGAGTAAC-3′) immediately up- and downstream of ICE*Ssu*SC117 (Fig. 3). The recipient S. suis P1/7 (ST1) and the donor S. suis SC117 (ST1314) were distinguished by multilocus sequence type (MLST).

**TABLE 1:** MICs of the *erm*(T)-carrying strains, the recipient strain S. suis P1/7, S. aureus, and their transformants

Strain	MIC (mg/L)^*a*^							
	FFC	ERY	LIN	CLI	GEN	CHL	TET	SPE
SC262	64	512	128	64	<1	32	32	2
SC117	16	512	128	64	2	32	64	>512
P1/7	<1	<1	<1	<1	2	<1	<1	16
P1/7+pSC262	<1	512	128	64	2	<1	<1	16
P1/7+ICE*Ssu*SC117	<1	512	128	64	2	<1	32	16
RN4220	4	<1	<1	<1	<1	4	<1	16
RN4220+pSC262	4	512	128	64	<1	4	<1	16
D39	<1	<1	<1	<1	2	<1	<1	2
D39+pSC262	<1	512	128	64	2	<1	<1	2

a FFC, florfenicol; ERY, erythromycin; LIN, lincomycin; CLI, clindamycin; GEN, gentamicin; CHL, chloramphenicol; TET, tetracycline; SPE, spectinomycin.

Furthermore, pSC262 was successfully transferred into the recipient strain S. aureus RN4220 (RN4220+pSC262) and Streptococcus pneumoniae D39 (D39+pSC262) by electrotransformation (19), confirmed by antimicrobial susceptibility testing (AST) (Table 1) and PCR. Compared with the recipient strains, the transformants RN4220+pSC262 and D39+pSC262 displayed elevated MICs of erythromycin, clindamycin, and lincomycin (Table 1), which indicated that an *erm*(T)-carrying plasmid can replicate and express macrolide-lincosamide resistance in heterologous hosts, including S. aureus and S. pneumoniae.

### Fitness cost analyses.

**FIG 4:**
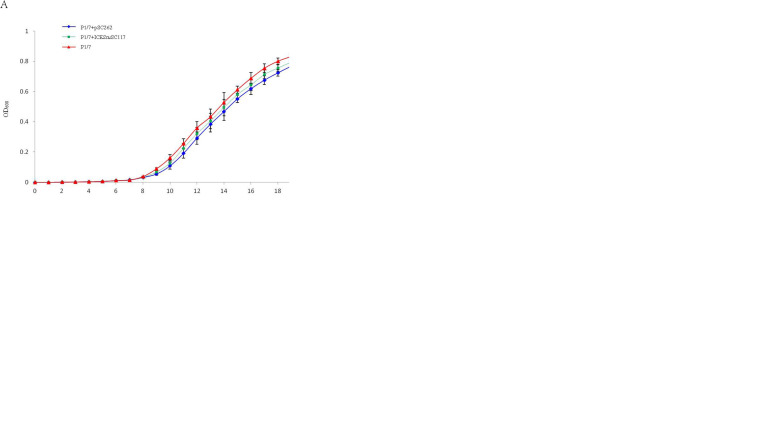
Fitness cost of pSC262 and ICE*Ssu*SC117 in S. suis in this study. (A) Comparison of the growth kinetics of strains S. suis P1/7, S. suis P1/7+pSC262, and S. suis P1/7+ICE*Ssu*SC117. (B) Growth competition between S. suis P1/7 and S. suis P1/7+pSC262. (C) Growth competition between S. suis P1/7 and S. suis P1/7+ICE*Ssu*SC117. The initial ratio of each strain in competition assay was 1:1.

The growth kinetics of P1/7, P1/7+pSC262, and P1/7+ICE*Ssu*SC117 in the antibiotic-free Todd-Hewitt broth (THB) were determined (Fig. 4A). The results showed no significant difference for the strains in the absence of selective pressure. However, competition experiments offered a more discriminative and precise measurement of fitness. From the second day on, an obvious decrease in the proportion of pSC262-carrying and ICE*Ssu*SC117-carrying strains was observed. At the 7th generation, the pSC262-carrying strain could not be detected (Fig. 4B), and the ICE*Ssu*SC117-carrying strain disappeared at the third generation (Fig. 4C). These findings suggests that the *erm*(T)-carrying strain had a fitness cost compared to S. suis P1/7, which will allow a susceptible strain to outcompete the resistant strain in the absence of a macrolide.

The acquisition of resistance is generally thought to be accompanied by a fitness cost to the bacterium (20). Spread and maintenance of a resistance gene are directly linked to the fitness cost associated with the gene expression. The constitutive expression of *erm*(T) in both plasmid pSC262 and ICE*Ssu*SC117 observed in this study will increase the fitness cost of *erm*(T)-carrying mobile genetic elements in these S. suis isolates, which may explain the low detection rate of the *erm*(T) gene in the S. suis population.

## MATERIALS AND METHODS

### Bacterial strains and AST.

A total of 237 nonduplicate S. suis strains were isolated and identified from individual diseased pigs in three provinces (Henan, Shanxi, and Guangdong) in China during 2010 to 2016. S. suis P1/7 and S. pneumoniae D39 served as the recipient strain in the transfer experiments (19). All strains were cultivated in THB at 37°C; the medium was supplemented with erythromycin (10 mg/L) for the selection of macrolide-resistant isolates. AST was performed by broth microdilution according to the recommendations given in the EUCAST breakpoint tables for interpretation of MICs and zone diameters, version 11.0 (21). The following antimicrobial agents were tested: florfenicol, erythromycin, lincomycin, clindamycin, gentamicin, chloramphenicol, tetracycline, and spectinomycin. Streptococcus pneumoniae ATCC 49619 served as the quality control strain. Simultaneously, the test for inducible clindamycin resistance in two *erm*(T)-positive S. suis isolates SC262 and SC117 was performed as described in CLSI document M100 to check whether the expression of *erm*(T) was inducible or constitutive (22).

### PCR analysis.

The *erm*(T) gene was detected in the S. suis strains by PCR using the primers *erm*(T)-fw, 5′-ATTGGTTCAGGGAAAGGTC-3′, and *erm*(T)-rv, 5′-TGGATGAAAGTATTCTCTAGGG-3′, and an annealing temperature of 53.5°C. The presence of circular intermediates in S. suis SC117 was detected by PCR using the primers ICE-circ-fw, 5′-TTGAACAGCCTAAAAGTGCCA-3′, and ICE-circ-rv, 5′-GTAAAGACCAAACAAAGACTCCAG-3′, and an annealing temperature of 59.0°C (Table S1).

### WGS analysis.

Whole-genome DNA of SC117 and SC262 was sequenced using the PacBio RS and Illumina MiSeq platforms (Shanghai Personal Biotechnology Co., Ltd., China). The PacBio sequence reads were assembled with HGAP4 and CANU (version 1.6) and then corrected by the Illumina MiSeq reads with pilon (version 1.22). The prediction of ORFs and their annotations were performed using Glimmer 3.0.

### Intraspecies transformation.

The transformation experiments were performed as described in a previous study (23). The peptide (GNWGTWVEE) was used as a pheromone for the transformation. The detailed protocols for the transformation were as follows. The recipient strain P1/7 was grown to exponential phase at 37°C under 5% CO_2_. Then, the logarithmic P1/7 strains were diluted 1:50 into Todd-Hewitt broth supplemented with yeast extract (THY) medium and grown to an optical density at 600 nm (OD_600_) between 0.035 and 0.058 at 37°C without shaking. The donor DNA (chromosomal DNA, 1 μg or plasmid, 1.2 μg) and synthetic peptide (250 μM) were added to the 100 μL bacterial samples. After 2 h of incubation at 37°C under 5% CO_2_, the samples were diluted, plated in THA plates with 5% sheep blood and 10 mg/L erythromycin, and incubated at 37°C overnight. Colonies were further confirmed by AST, 16s RNA sequencing, and MLST following harmonized protocols (http://pubmlst.org/) (Table S1).

### Interspecies transformation.

To investigate the replication ability of the *erm*(T)-carrying plasmid pSC262 in heterologous hosts, transformation assays were performed. Plasmid DNA was extracted by using the Qiagen plasmid extraction midi kit (Qiagen, Hilden, Germany) according to the following procedure. After the S. suis SC262 was suspended in buffer P1, lysozyme was added at a final concentration of 20 g/mL, and the mixture was incubated for 2 h at 37°C before buffer P2 was added. Transfer of the purified plasmid DNA was attempted with S. aureus RN4220 and S. pneumoniae D39 by electrotransformation. The transformants were selected on brain heart infusion (BHI) agar supplemented with 10 μg/mL erythromycin.

### Fitness cost experiments.

The growth kinetics of S. suis P1/7 and the two transformants P1/7+pSC262 and P1/7+ICE*Ssu*SC117 were determined. Cultures were grown for 24 h at 160 rpm and 37°C, and the absorbance at 600 nm was measured every hour.

The fitness cost of the plasmid pSC262 was determined by three independent competition experiments between P1/7 and P1/7+pSC262, and the fitness cost of the ICE*Ssu*SC117 was determined between P1/7 and P1/7+ICE*Ssu*SC117, as described previously (24). Strains were grown in THB for 16 h at 37°C. Then, 1 × 10^8^ CFU of P1/7 was mixed with 1 × 10^8^ CFU of P1/7+pSC262 or P1/7+ICE*Ssu*SC117, respectively. The mixtures were grown at 37°C and 160 rpm and diluted at 1:100 to fresh THB every 12 h. Before every dilution, samples were taken and plated onto antibiotic-free and erythromycin-containing THA plates simultaneously. The number of colonies growing on erythromycin plates was the number of drug-resistant bacteria in the mixed culture system. The number of colonies on the antibiotic-free plate minus the number of colonies on the erythromycin plate is the number of susceptible bacteria in the mixed culture system.

### Data availability.

The sequences of the plasmid pSC262 and the ICE*Ssu*SC117, determined in this study, have been deposited in GenBank under accession numbers CP06178 and MW026423, respectively.
