# Supplementary material for: erm(T)-Mediated Macrolide-Lincosamide Resistance in Streptococcus suis
Source: Microbiol Spectr. 2022 Jan 12;10(1):e01657-21. doi: 10.1128/spectrum.01657-21 (PMC8754144; doi:10.1128/spectrum.01657-21)
Supplement: SUPPLEMENTAL FILE 1 — Supplemental material. Download Spectrum01657-21_Supplemental_File_1.docx, DOCX file, 0.02 MB [file spectrum01657-21_supplemental_file_1.docx]

**Supplemental File 1**

**TABLE S1.** Oligonucleotide primer pairs used

| Category and gene | Primer designation | Sequence (5'-3') | Annealing tem (^o^C) | Product size(bp) |
| --- | --- | --- | --- | --- |
| *erm*(T) | *erm*(T)-fw  *erm*(T)-rv | ATTGGTTCAGGGAAAGGTC  TGGATGAAAGTATTCTCTAGGG | 53.5 | 387 |
| ICE-circ | ICE-circ-fw  ICE-circ-rv | TTGAACAGCCTAAAAGTGCCA  GTAAAGACCAAACAAAGACTCCAG | 59.0 | 2537 |
| 16s RNA | 16s RNA-fw  16s RNA-rv | AGAGTTTGATCCTGGCTCAG  ACGGCTACCTTGTTACGACTT | 56.0 | 1466 |
| *aroA* | *aroA*-fw  *aroA*-rv | TTCCATGTGCTTGAGTCGCTA  ACGTGACCTACCTCCGTTGAC | 50.0 | 366 |
| *cpn* | *cpn*-fw  *cpn*-rv | TTGAAAAACGTRACKGCAGGTGC  ACGTTGAAIGTACCACGAATC | 52.0 | 318 |
| *dpr* | *dpr*-fw  *dpr*-rv | CGTCTTTCAGCCCGCGTCCA  GACCAAGTTCTGCCTGCAGC | 50.0 | 336 |
| *gki* | *gki*-fw  *gki*-rv | GGAGCCTATAACCTCAACTGG  AAGAACGATGTAGGCAGGATT | 55.0 | 321 |
| *mutS* | *mutS*-fw  *mutS*-rv | CGCAGAGCAGATGGAAGATCC  CCCATAGCTGTTTTGGTTTCATC | 50.0 | 339 |
| *recA* | *recA*-fw  *recA*-rv | TATGATGAGTCAGGCCATG  CGCTTAGCATTTTCAGAACC | 50.0 | 354 |
| *thrA* | *thrA*-fw  *thrA*-rv | GATTCAGAACGTCGCTTTGT  AAGTTTTCATAGAGGTCAGC | 52.0 | 336 |
