# Supplementary material for: erm(T)-Mediated Macrolide-Lincosamide Resistance in Streptococcus suis
Source: Microbiol Spectr. 2022 Jan 12;10(1):e01657-21. doi: 10.1128/spectrum.01657-21 (PMC8754144; doi:10.1128/spectrum.01657-21)
Supplement: SUPPLEMENTAL FILE 2 — Supplemental material. Download Spectrum01657-21_Supplemental_File_2.docx, DOCX file, 0.25 MB [file spectrum01657-21_supplemental_file_2.docx]

**Supplemental File 2**


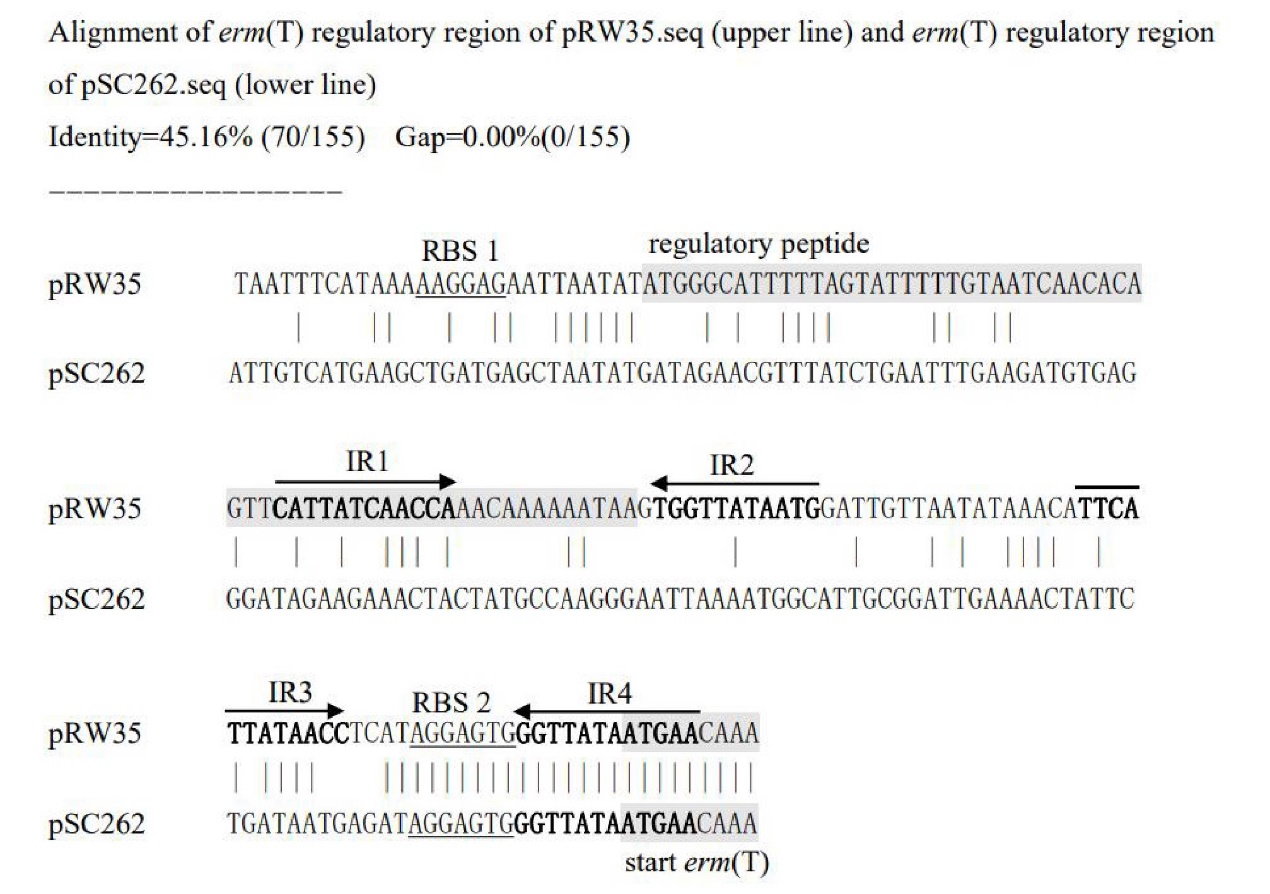


**FIG S1.** Comparison of the *erm*(T) regulatory region of pSC262 with those of plasmids pRW35 (EU192194). Identical bases are indicated by vertical bars. The two pairs of IR sequences, IR1-IR2 and IR3–IR4, are marked by arrows and displayed in bold letters. Ribosomal binding sites RBS 1 and RBS 2 are underlined. The reading frame for the regulatory peptide and the 5′-terminal part of the *erm*(T) gene are indicated by grey shading.
