# Supplementary material for: erm(T)-Mediated Macrolide-Lincosamide Resistance in Streptococcus suis
Source: Microbiol Spectr. 2022 Jan 12;10(1):e01657-21. doi: 10.1128/spectrum.01657-21 (PMC8754144; doi:10.1128/spectrum.01657-21)
Supplement: SUPPLEMENTAL FILE 3 — Supplemental material. Download Spectrum01657-21_Supplemental_File_3.docx, DOCX file, 0.3 MB [file spectrum01657-21_supplemental_file_3.docx]

**Supplemental File 3**


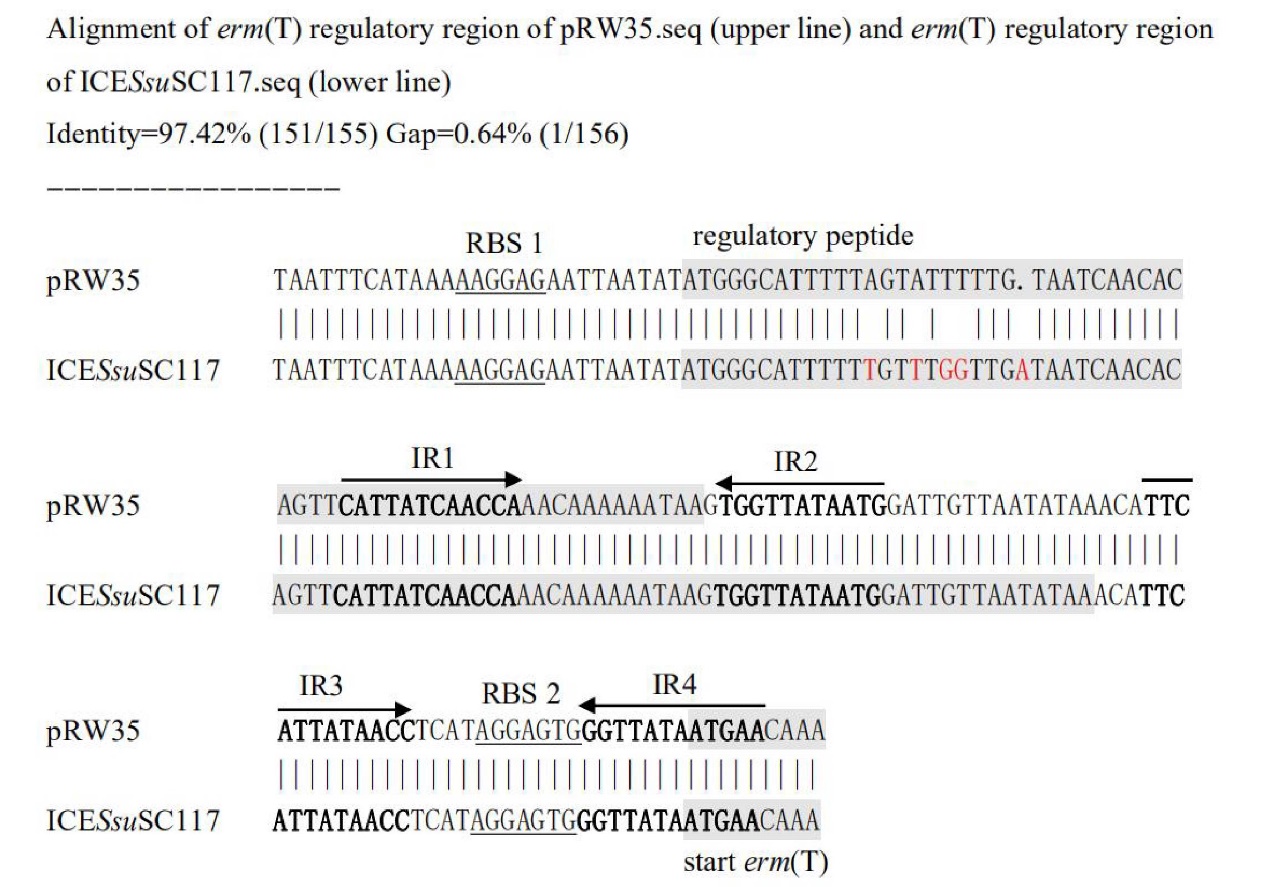


**FIG S2.** Comparison of the *erm*(T) regulatory region of ICE*Ssu*SC117 with those of plasmids pRW35 (EU192194). The base alignment mark is the same as Figure S1.
